# Beneficial Effects of Melatonin on the In Vitro Maturation of Sheep Oocytes and Its Relation to Melatonin Receptors

**DOI:** 10.3390/ijms18040834

**Published:** 2017-04-17

**Authors:** Xiuzhi Tian, Feng Wang, Lu Zhang, Changjiu He, Pengyun Ji, Jing Wang, Zhenzhen Zhang, Dongying Lv, Wusiman Abulizi, Xuguang Wang, Zhengxing Lian, Guoshi Liu

**Affiliations:** 1 National Engineering Laboratory for Animal Breeding, Key Laboratory of Animal Genetics and Breeding of the Ministry of Agriculture, Beijing Key Laboratory for Animal Genetic Improvement, College of Animal Science and Technology, China Agricultural University, Beijing 100193, China; tian7550@163.com (X.T.); vicent007@126.com (F.W.); df765@sina.com (L.Z.); chungjoy@cau.edu.cn (C.H.); jipengyun1989@126.com (P.J.); caylajing@163.com (J.W.); taoyaerxl@gmail.com (Z.Z.); suxingdemogu@icloud.com (D.L.); lianzhx@cau.edu.cn (Z.L.); 2 College of Animal Science, Xinjiang Agricultural University, Urumqi 830052, China; abulizi68@126.com (W.A.); wang_xuguang@126.com (X.W.)

**Keywords:** melatonin, oocyte, meioctic maturation, MT1/MT2, sheep

## Abstract

(1) Background: The binding sites of melatonin, as a multifunctional molecule, have been identified in human, porcine, and bovine samples. However, the binding sites and mechanisms of melatonin have not been reported in sheep; (2) Methods: Cumulus–oocyte complexes (COCs) were cultured in TCM-199 supplemented with melatonin at concentrations of 0, 10^−3^, 10^−5^, 10^−7^, 10^−9^, and 10^−11^ M. Melatonin receptors (MT1 and MT2) were evaluated via immunofluorescence and Western blot. The effects of melatonin on cumulus cell expansion, nuclear maturation, embryo development, and related gene (*GDF*9, *DNMT*1, *PTX3*, *HAS2*, and *EGFR*) expression were investigated. The level of cyclic adenosine monophosphate (cAMP) and cyclic guanosine monophosphate (cGMP) were evaluated in oocytes and cumulus, respectively; (3) Results: Both MT1 and MT2 were expressed in oocytes, cumulus cells, and granulosa cells. Melatonin with a concentration of 10^−7^ M significantly enhanced the rates of nuclear maturation, cumulus cells expansion, cleavage, and blastocyst. Melatonin enhanced the expression of *BMP*15 in oocytes and of *PTX3*, *HAS2*, and *EGFR* in cumulus cells. Melatonin decreased the cAMP level of oocytes but enhanced the cGMP level in oocytes and cumulus cells; (4) Conclusion: The higher presence of MT1 in GV cumulus cells and the beneficial effects of melatonin indicated that its roles in regulating sheep oocyte maturation may be mediated mainly by the MT1 receptor.

## 1. Introduction

Compared to in-vivo-derived embryos, the efficiency of in vitro embryo development is still low [1], which is mainly because the in vitro handling and culture conditions are far from perfect when oocytes and embryos are exposed to in vivo conditions. To solve this problem, many efforts have been made. Recently, the focus has been on melatonin, which is a potent antioxidant and free radical scavenger [2,3] due to its beneficial effects on oocytes maturation and embryo development [4,5,6,7,8,9,10,11,12,13,14]. Melatonin is well known for its physiological functions in seasonal reproduction, energy metabolism, and thermo-regulation in mammals [15]. Melatonin and its receptors are present in reproductive organs [16] in which both of them play roles in the functions of testes and ovaries [17], and are able to modify the morphology and steroidogenesis capacity [18,19,20,21]. Melatonin stimulates ovarian activity and promotes estrous cyclicity and gonadal atrophy depending on photoperiodic conditions [22]. Melatonin also regulates folliculogenesis and ovulation [23] by acute suppression of luteinizing hormone secretion [24]. Additionally, it was reported that melatonin affects the axis by directly binding to granulosa cells in the ovary [25]. Melatonin binding sites have been identified not only in granulosa cells of human preovulatory follicles [25,26] but also in porcine cumulus and granulosa cells [3], bovine oocytes, and cumulus cells [9]. However, the exact mechanisms of melatonin on the IVM (in vitro maturation) of sheep oocytes and in vitro embryonic development is still elusive.

The resumption of oocyte meiotic maturation and germ vesicle breakdown (GVBD) after gonadotropin stimulation is mediated mainly through increasing intracellular cyclic adenosine monophosphate (cAMP) level in the cumulus cells [27]. Elevated cAMP in cumulus cells rather than in the oocytes is also known to stimulate MAPK activation [28,29,30]. It has been reported that the supplementation of the cAMP promotor in IVM medium inhibited the oocyte meiotic mitosis of mice and humans [31,32], but only temporarily inhibited the oocyte meiotic mitosis of cows [33,34]. Many studies have demonstrated that supplementation of cAMP mediator improved the development of oocytes [32,34,35].

Based on the evidence mentioned above, melatonin exhibited its specific effects upon sheep oocyte maturation either via its direct antioxidant activity or mediated by its receptor activation. The specific objectives of the current study were (i) to determine whether MT1 and MT2 are expressed in sheep oocytes, cumulus cells, and granulosa cells; (ii) to investigate what the optimal melatonin concentration is to promote in vitro oocyte maturation and embryo development; and (iii) to assess the effect of melatonin on the expression of embryo development-related genes in cumulus cells and oocytes; (iv) to investigate the effect of the antagonist of melatonin and the effect of melatonin on cAMP and cGMP (cyclic guanosine monophosphate) in oocytes and cumulus cells.

## 2. Results

### 2.1. Melatonin Receptors in Sheep Oocytes, Cumulus Cells, and Granulosa Cells

**Figure 1:**
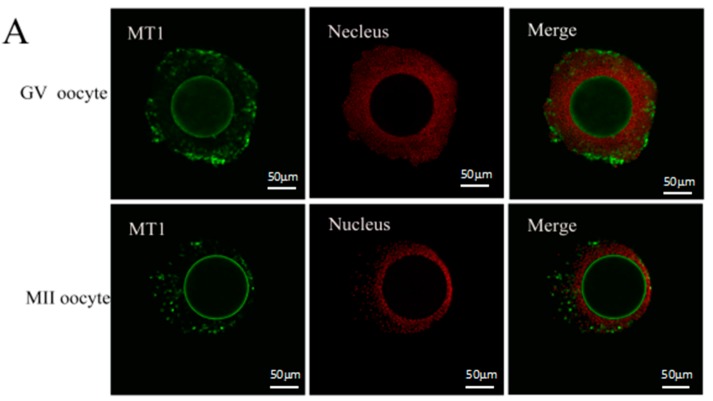

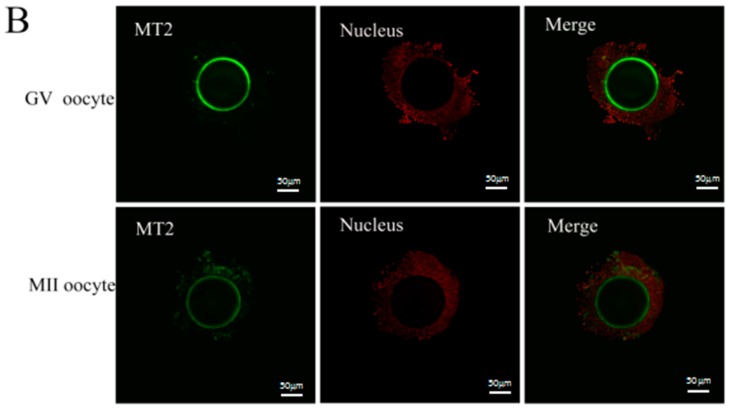
Assayed the melatonin receptor with immunofluorescence. The cellular localizations of MT1/MT2 were identified by confocal analysis. Sheep COCs were incubated with the MT1/MT2 antibody (red) followed by Alexa-488-conjugated donkey anti-goat IgG (green). (**A**) images of MT1 receptor; (**B**) images of MT2 receptor. GV: germinal vesicle stage, MII: metaphase II stage. Scale bar = 50 µm.

**Figure 2:**
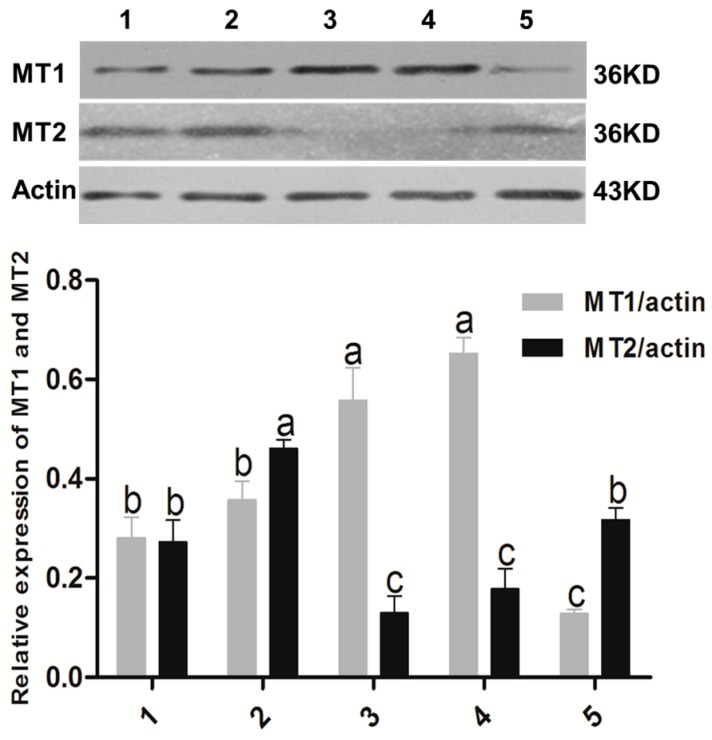
The expression of MT1 and MT2 identified with Western blot method. 1. Oocyte in GV stage; 2. Oocyte in MII stage; 3. Granulosa cells; 4. Cumulus cells in GV stage; 5. Cumulus cells in MII stage. The superscript different letters (a–c) represent a significant difference in the same column (*p* < 0.05).

The expression of MT1 and MT2 receptor was confirmed by using immunofluorescence and Western blot analysis with specific antibodies in GV (germinal vesicle) and MII (metaphase II) oocytes, and GV and MII cumulus cells in the granulosa cells. The expression level of MT1 receptors was significantly higher in GV cumulus cells and granulosa cells compared to that in three other cell types, while the expression level of the MT2 receptor in MII oocytes was highest among all cell types (Figure 1 and Figure 2).

### 2.2. The Effect of Melatonin on Cumulus Expansion and Nuclear Maturation of Oocytes

**Figure 3:**
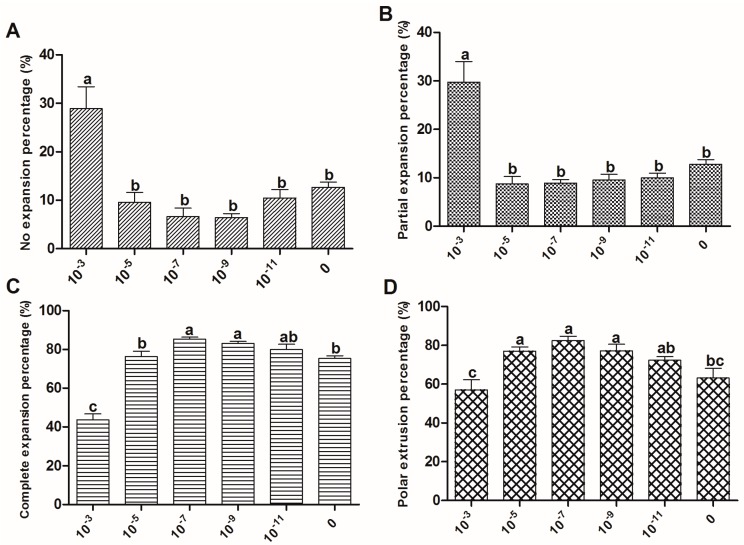
Effects of melatonin on cumulus cell expansion and oocyte nuclear maturation. Maturation rate of oocytes treated with melatonin (0, 10^−3^, 10^−5^, 10^−7^, 10^−9^, and 10^−11^ M) for 24 h, cumulus cell expansion, and polar body extrusion were analyzed, respectively. (**A**) No expansion percentage; (**B**) Partial expansion percentage; (**C**) Complete expansion percentage; (**D**) Polar extrusion percentage. Data are expressed as percentage ± SEM from six independent experiments (>30 oocytes per treatment per experiment). Bars with different letters (a–c) represent significantly different (*p* < 0.05).

A significant increase in the percentage of oocytes at MII stage was observed in cumulus-oocyte complexes (COCs) supplemented with 10^−5^~10^−9^ M melatonin. The most efficient concentration of melatonin for this purpose is 10^−7^ M (Figure 3). The result agreed with the cumulus expansion at the same melatonin concentration (10^−7^ M) (85.3% ± 1.01% vs. 75.3% ± 1.45%, *p* < 0.05). In contrast, the adverse effects of the extremely high concentration of melatonin (10^−3^ M) were observed (Figure 3).

### 2.3. The Effect of Melatonin on Embryo Development

**Figure 4:**
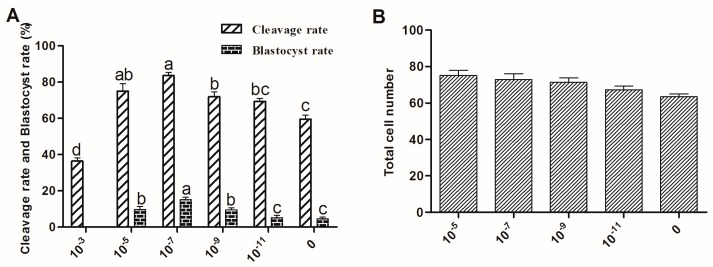
Effect of melatonin on the development of parthenogenetic activation sheep oocytes. Embryos were acquired by parthenogenetic activation oocytes that were cultured in the maturation medium contained different concentrations of melatonin. (**A**) cleavage rate and blastocyst rate; (**B**) cell number/blastocyst. The superscript different letters (a–d) represent a significant difference in the same column (*p* < 0.05).

The cleavage rate of oocytes at 48 h after parthenogenetic activation and the blastocyst rate at Day 8 of incubation were recorded. Oocytes treated with 10^−7^ M melatonin had a significantly higher cleavage rate (83.6% ± 1.75% vs. 59.5% ± 2.25% in control, *p* < 0.05) and blastocyst rate (15.0% ± 1.45% vs. 4.5% ± 0.94% in control, *p* < 0.05) than those of the oocytes in the control groups. Oocytes recovered from 10^−5^~10^−11^ M melatonin-treated group developed into blastocysts, which had higher total cell number than that in the control group and in the 10^−3^ M melatonin-treated group (Figure 4).

### 2.4. The Effect of Melatonin on the Embryo Development-Related Genes Expression in Oocytes and Cumulus Cells

**Figure 5:**
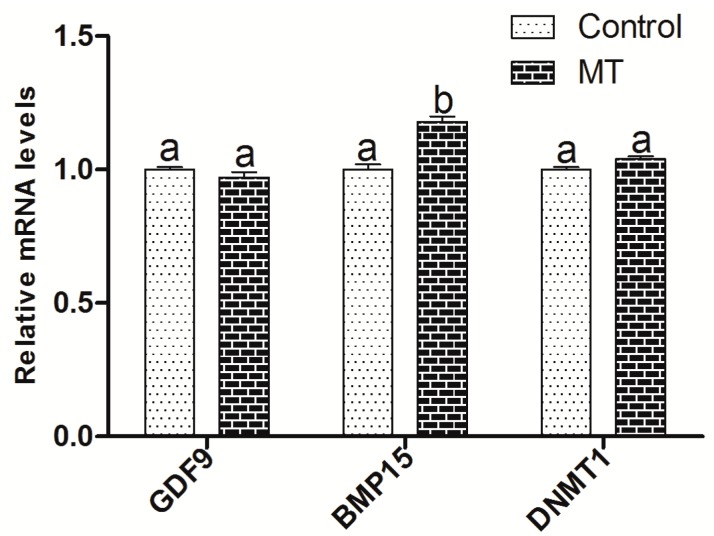
Effects of 10^−7^ M melatonin on the gene expression in sheep oocytes. The genes include the oocyte-secreted factors (*GDF9* and *BMP15*) and DNA methyltransferase 1 (*DNMT1*). The letters a and b identify statistically significant differences (*p* < 0.05).

**Figure 6:**
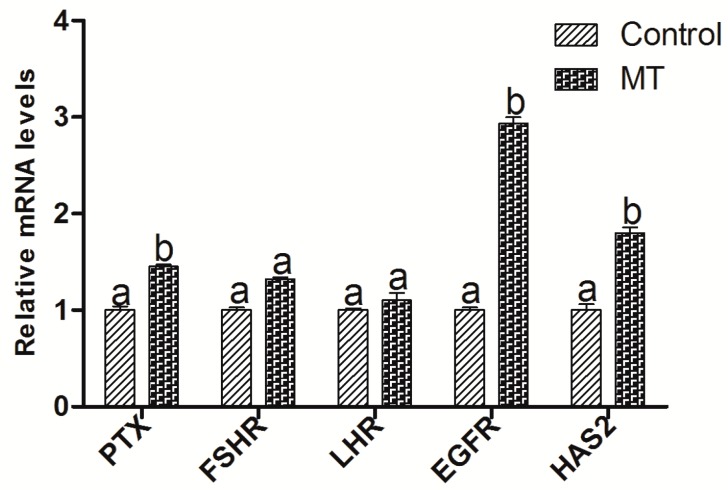
Effects of 10^−7^ M melatonin on the gene expression of cumulus cells expansion. The genes include *PTX3*, *HAS2*, *LHR*, *FSHR*, and *EGFR*. The letters (a,b) identify statistically significant differences (*p* < 0.05).

After IVM for 8 h in the absence or presence of 10^−7^ M melatonin, oocytes and cumulus cells were collected, respectively, for measuring the expression of the embryo-development-related genes. The results showed that melatonin treatment had a limited effect on these gene expressions (mRNAs), including *GDF9* (growth differentiation factor) and *DNMT1* (DNA methyltransferase 1) in oocytes, but increased the expression of *BMP15* when compared with the control group (*p* < 0.05) (Figure 5). Gene expressions of *PTX3* (pentraxin 3), *HAS2* (hyaluronan synthase 2), and *EGFR* (epidermal growth factor receptor) in cumulus cell expansion were upregulated by melatonin treatment (*p* < 0.05), while melatonin had no significant effects on the expressions of *FSHR* (follicle stimulating hormone receptor) or *LHR* (luteinizing hormone receptor) (Figure 6).

### 2.5. MT Promoted Maturation of Sheep Oocytes through Melatonin Receptors

**Figure 7:**
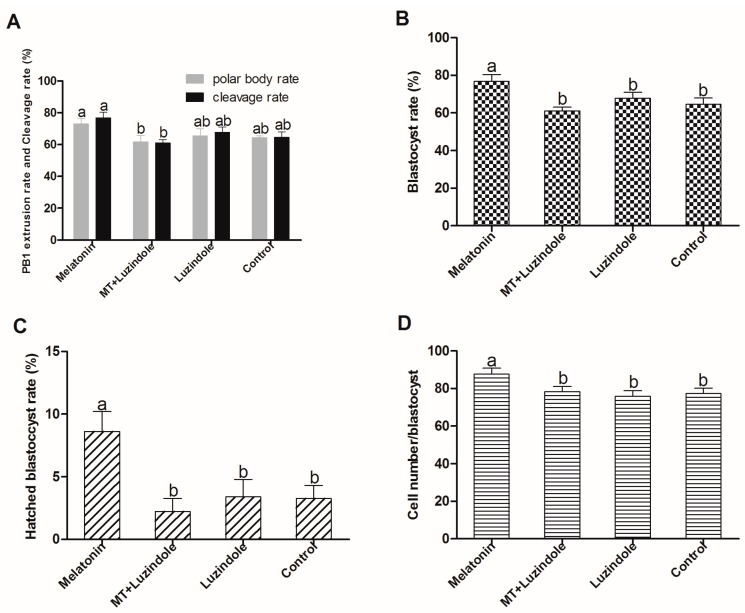
Effects of melatonin receptor antagonist luzindole (10^−^^6^ M) on the in vitro maturation of sheep oocytes. (**A**) PB1 extrusion rate and cleavage rate; (**B**) Blastocyst rate; (**C**) Hatched blastocyst rate; (**D**) Cell number/blastocyst. The numbers of cultured oocytes for each group varied from 200 to 250, respectively. Different superscript letters (a,b) in each column identify statistical significant differences (*p* < 0.05).

A total of 1276 oocytes were used in six replicates to identify the potential associations of melatonin receptor activation and sheep oocyte maturation. The results showed that the rates of polar body, cleavage, blastocyst, and hatched blastocyst were significantly higher in the melatonin-treated group than that in the group treated with melatonin plus luzindole (10^−^^6^ M). However, the polar body rate and cleavage rate of the MT group was not significantly different from the luzindole group or the control group (Figure 7).

### 2.6. The Effect of Melatonin on cAMP and cGMP Concentration in Oocytes and Cumulus Cells

**Figure 8:**
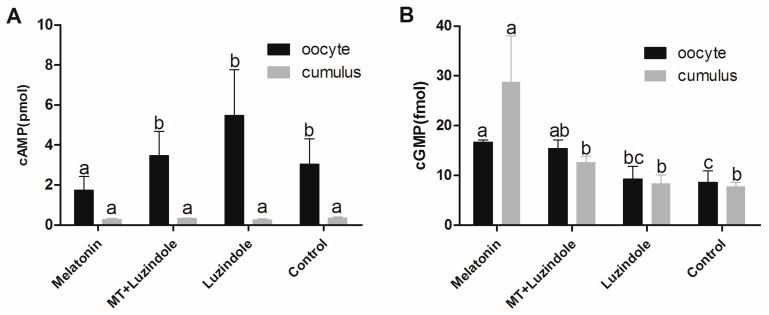
Intracellular cAMP concentration of oocytes and cumulus cells in different treatments. (**A**) The concentration of cAMP per oocyte or cumulus cell; (**B**) The concentration of cGMP per oocyte or cumulus cell. Data are from five independent repeats and are shown as mean ± SEM. The superscript different letters (a,b) represent a significant difference in the same column compared to the control (*p* < 0.05).

This experiment was designed to investigate the effect of 10^−7^ M melatonin and 10^−^^6^ M melatonin receptor antagonist luzindole on cAMP and cGMP concentration in oocytes and cumulus cells. The results showed that the concentration of cAMP in oocytes with melatonin treatment was significantly lower than that in other groups including the groups treated with luzindole and melatonin plus luzindole, respectively, and the control group; however, there were no differences in cAMP in the cumulus cells. The concentration of cGMP in both oocytes and cumulus cells with melatonin treatment was higher than that in the other groups (Figure 8).

## 3. Discussion

This study for the first time identified that the specific melatonin receptor (MT1 and MT2) were not only expressed in sheep granulosa cells, but also in cumulus cells and oocytes in GV and MII stages (Figure 1 and Figure 2). The Western blot results showed that there was a high expression of MT1 in cumulus cells, while the expression of MT2 was quite low. Based on the observations, we speculated that some effects of melatonin on sheep COCs were likely to be mediated by melatonin receptors. The identification of MT1 and MT2 in granulosa cells, cumulus cells, and oocytes in sheep were accordance with the observations of El-Raey et al. found in cows [9], and it confirmed previous reports that melatonin was important for the regulation of reproduction in animals [36]. The expression levels of melatonin receptors in granulosa cells, cumulus cells, and oocytes varied at different stages, which implied that the receptor activation has its unique functions at different stages of embryo development.

Mammalian cumulus cells play a very important role during oocyte growth and maturation. Cumulus cells expansion is considered as an important marker for oocyte maturation [37,38] and thus is essential for fertilization, subsequent cleavage, and blastocyst development [39]. They are known to supply nutrients [40,41,42] and messenger molecules for oocyte development [43], and to mediate the effects of steroid hormones on oocytes [44]. It was confirmed that melatonin supplementation to IVM medium had a significant effect on sheep cumulus cells expansion and polar body extrusion; melatonin at a concentration of 10^−7^ M was particularly optimal for nuclear maturation and oocyte quality maintenance (Figure 3 and Figure 4). The results were consistent with our previous report on bovine oocytes [14]. The similar beneficial effects of melatonin on cumulus cells expansion were also reported in porcine oocytes [45]. As an antioxidant, melatonin protected cumulus cells against apoptosis [45,46,47] and enhanced their expansion [45]; moreover, we found that these effects of melatonin can also be mediated by its receptor activation.

GDF9 and BMP15 are members of the transforming growth factor β-superfamily, which are secreted from oocytes during folliculogenesis. Oocytes regulates cumulus cell proliferation, apoptosis, metabolism, and expansion by secreting these two paracrine factors [48]. In mammals, BMP15 is predominantly produced by oocytes and exerts important regulatory functions within the ovary, such as promoting early folliculogenesis, preventing premature luteinization, and enhancing cumulus cell expansion. It was reported that BMP-15 could inhibit precocious oocyte maturation in zebrafish, and this action reduced oocyte quality and subsequent ovulation and fertilization [49]. A high level of GDF9 in the follicular fluid is correlated with oocyte nuclear maturation and embryo quality [50]. The current study demonstrated that melatonin in a maturation medium had limited effect on the expression of *GDF9* and *DNMT1* in oocytes, but enhanced the expression of *BMP15* in oocytes and *PTX3*, *HAS2*, and *EGFR* in cumulus cells (Figure 5 and Figure 6). This was conflict with previous report in which the expression of *GDF9* and *DNMT1* in oocyte was increased with melatonin treatment [14]. It is found that MT's binding of the receptors has a more notable effect in cumulus cells than in oocytes, so the alterations of gene expression caused by melatonin mainly occurred in the cumulus cells. Taken together, it is possible that MT may improve in vitro COCs maturation in sheep mainly via the MT1 receptor.

**Figure 9:**
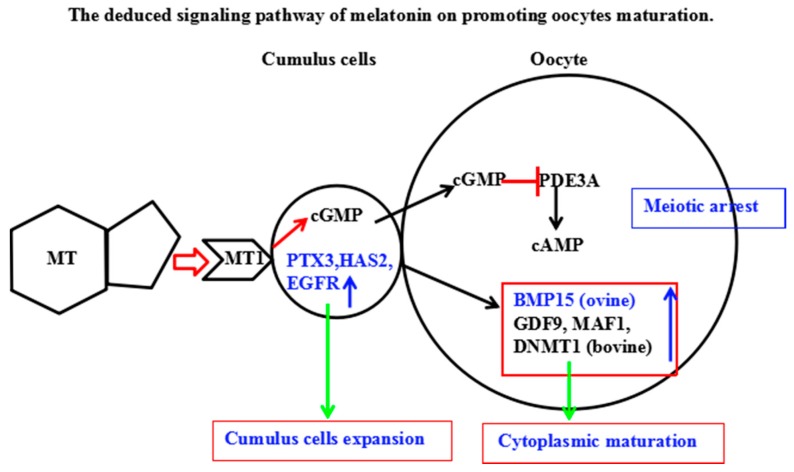
Schematic representation of melatonin roles on promoting oocytes maturation. PDE: phosphodiesterase; cGMP: cyclic guanosine monophosphate; cAMP: cyclic adenosine monophosphate. Red arrow: promoting action; blue arrow: gene expression improved; green arrow: improved gene expression resulted in cumulus cells expansion; red T bar: inhibiting effect.

Melatonin has been the agent of choice for improving oocyte quality in women who failed pregnancy due to poor oocyte quality [51]. Increased melatonin concentration in the follicular fluid was reported to reduce the level of lipid peroxidation, which caused DNA damage in oocytes [51,52]. Melatonin was reported to enhance meiotic maturation of porcine [3], buffalo [52], and mouse oocytes in in vitro conditions [53]. Moreover, melatonin supplementation to IVM medium rescued mouse oocytes from meiotic arrest induced by dbcAMP or hypoxanthine treatment, regardless of the concentration of oxygen [53], suggesting its role in meiotic resumption. Our results showed, for the first time, that melatonin and its receptor inhibitor, luzindole, supplemented into IVM medium greatly affected oocyte cAMP. It was reported that melatonin inhibited adenylate cyclase activity via membrane-bound G protein-coupled MT1 and MT2 receptor [54]. In this study, we observed that melatonin treatment increased the level of cAMP in COCs, which is similar to the previous report [55], which indicated that melatonin could temporarily inhibit the oocyte meiotic mitosis by prolonging cAMP modulated oocyte maturation, and thus increased oocyte cumulus cells gap-junctional communication and subsequently improved the embryo quality and its development. In addition, melatonin-treated COCs had higher levels of cGMP in oocytes and cumulus cells. A high level of cGMP in cumulus cells affects phosphodiesterase (PDE), which is a regulator of cAMP production in oocytes and granulosa [33]. PDE is also an inhibitor of PDE3/4 and the inhibition of PDE3/4 improves the embryo development of the bovine IVM oocytes. Therefore, melatonin treatment delays the GVBD of oocytes and prolongs the communication time of oocytes and granulosa cells during the period of meiosis. Both facilitate the exchange of regulatory molecules and metabolic substances between oocytes and granulosa cells, thereby improving the quality of oocytes. In other words, via its MT1 receptor activation, melatonin increases the concentrations of cAMP in oocytes and cGMP in cumulus cells and promotes the cytoplasmic maturation and the ability of oocyte development. A signal transduction pathway for melatonin promoting oocytes maturation is summarized in Figure 9.

The level of cAMP in oocyte is crucial for the maintenance of meiotic arrest. Inhibition of oocyte cAMP-phosphodiesterase (PDE3A) activity results in sustaining elevated cAMP level [56]. Cyclic GMP in cumulus cells will diffuse into the oocyte via gap junctions and it inhibits PED3A activity and cAMP hydrolysis. These events lead to meiotic arrest [57,58]. MT also upregulates the expression of *PTX3*, *HAS2*, and *EGFR* in cumulus cells to promote cumulus cells expansion as well as the expression of *BMP15*, *GDF9*, *DNMT1*, and *MAF1* in oocytes to advance oocyte maturation.

## 4. Materials and Methods

### 4.1. Chemicals

All chemicals and media, except otherwise specified, were purchased from Sigma Chemical Co. (St. Louis, MO, USA) and Gibco (Grand Island, NY, USA).

### 4.2. Animal Studies

The study was carried out in strict accordance with the protocol approved by the Animal Welfare Committee of China Agricultural University (Permission Number: SYXK(Beijing)2015002; The period of valid days: 22 September 2015–22 September 2020).

### 4.3. Ovary Collection and Cumulus–Oocyte Complex Aspiration

Sheep ovaries were collected from local abattoir and transported to the laboratory in sterile saline solution supplemented with penicillin (100 IU/mL) and streptomycin (100 IU/mL) at 25–30 °C within 3 h after slaughter, and they were washed in a sterile saline solution immediately after arrival. COCs were recovered from the ovary with a blade in a culture dish with a 90 mm diameter. Hepes-buffered tissue culture medium-199 (HTCM-199) supplemented with 0.1% polyvinyl pyrrolidine alcohol (PVA), 25 IU/mL heparin, and penicillin, as well as streptomycin, was used for handling oocytes. Only aspirated COCs with compact and intact cumulus and homogeneous cytoplasm were selected under a stereomicroscope for experiments.

### 4.4. In Vitro Maturation of Oocytes 

The tissue culture medium-199 (TCM-199) supplemented with Napyruvate (2.5 mM), l-glutamine (1.0 mM), penicillin (100 IU/mL), streptomycin (100 IU/mL), 10% fetal bovine serum, and cysteamine (0.1 mM) was used as the basal culture medium. Maturation medium was modified from basal culture medium by adding 100 ng/mL EGF, 10 μg/mL FSH, 10 μg/mL LH, and 1 μg/mL estradiol-17β. Melatonin and luzindole (an antagonist of melatonin receptors) were dissolved in DMSO at concentrations of 1 and 0.1 M separately and then stored at −20 °C as stock solutions. The stock solutions were diluted in TCM-199 to their final concentrations (melatonin: 10^−3^, 10^−5^, 10^−7^, 10^−9^, 10^−11^ M; luzindole: 10^−6^ M) immediately before use. COCs were cultured in groups of 90 in 700 μL droplets in maturation medium in 4-well plates at 38.5 °C with 5% CO_2_ in humidified air for 24 h.

### 4.5. Parthenogenetic Activation

Parthenogenetic activation (PA) of sheep oocytes was performed according to Hosseini et al. [59] with modification. Ovine oocytes were denuded after 24 h of culturing, and the oocytes with a polar body were then chosen for PA. Oocytes were first exposed to 5 mM ionomycin in HTCM-199 containing 0.1% PVA for 5 min. They were then carefully washed in HTCM-199 containing 0.1% PVA and incubated with 2 mM 6-dimethyl amino purine for 4 h. Oocytes were then washed in modified synthetic oviduct fluid (mSOF) and cultured in group of 20 oocytes in 60 μL droplets of mSOF without serum and glucose for 2 days. These were further treated with 10% fetal serum (10%) and glucose (1.5 mM) for 6 days (sequential mSOF). The cleavage rate was determined on the 2nd day and the blastocyst rate on the 8th day after PA.

### 4.6. Detection of Melatonin Receptors in COCs by Immunofluorescence

COCs were fixed with 4% paraformaldehyde for 45 min, washed thrice in dulbecco's phosphate buffered saline (DPBS) containing 0.1% PVA (10 min per wash), and finally incubated with blocking reagent (DPBS containing 1% BSA) at 4 °C overnight. The blocked oocytes were washed thrice in 0.1% PVA-DPBS (20 min per wash) and incubated with a primary antibody (MT1: SC-13186, 1:200 dilution, Santa Cruz Bio Inc., Santa Cruz, CA, USA; MT2: SC-13177, 1:200 dilution, Santa Cruz Bio Inc., Santa Cruz, CA, USA) at 37 °C for 2 h in the dark, washed thrice in DPBS containing 0.1% PVA (20 min per wash), and then incubated with a secondary antibody (Alexa Fluor 594 Molecular Probes, 1:1000 dilution, Life Technologies, Carlsbad, MA, USA) at 37 °C for 1 h in the dark. Finally, the samples were washed thrice in DPBS containing 0.1% PVA, stained with PI, and examined with confocal laser microscopy (Olympus FV1000, Tokyo, Japan).

### 4.7. Detection of Melatonin Receptors by Western Blotting

Granulosa cells, GV oocytes, and GV cumulus cells were collected immediately after the COCs were aspirated, and after maturing for 24 h, MII oocytes and cumulus cells were collected. All collected samples were washed thrice with PBS and frozen at −80 °C until use. Proteins were subjected to SDS-PAGE with 12% polyacrylamide gel and then transferred to a nitrocellulose membrane (BioTraceNT, Pall Corporation, Ann Arbor, MI, USA) for 2.5 h under a 300 mA electric current. After blocking the membrane with 5% skimmed milk overnight at 4 °C, the membrane was incubated with a primary antibody (MT1: SC-13186, 1:300 dilution, Santa Cruz Bio Inc., Santa Cruz, CA, USA; MT2: SC-13177, 1:150, dilution, Santa Cruz Bio Inc., Santa Cruz, CA, USA; actin: AC-15, 1:1000, ab6276, Amyjet Scientific Inc., Abcam, Cambridge, UK). To visualize the protein-bound antibodies, we used horseradish peroxidase (HRP)-conjugated anti-goat IgG (1:1000 dilution, Santa Cruz Bio Inc., Santa Cruz, CA, USA), followed by a detection procedure using an ECL detection kit (Amersham, Buckinghamshire, UK) according to the manufacture’s instructions.

### 4.8. Assessment of Cumulus Cells Expansion and Polar Body Extrusion

The degree of cumulus cells expansion was assessed under a stereomicroscope after 24 h of maturation subjectively as not expanded, partially expanded (the outer layer of cells was loosened), or fully expanded (all cumulus cells were loosened). After assessing the cumulus cells expansion, the COCs were denuded and the polar bodies were counted.

### 4.9. Assessment of Embryo Quality

The quality of blastocysts was assessed by Hoechst 33342 staining for 10 min. After rinsing in DPBS containing 0.1% PVA medium, blastocysts were mounted on a clean glass slide, covered with a coverslip, and examined under an inverted microscope (Nikon Corp., Tokyo, Japan) equipped with epifluorescence.

### 4.10. RNA Isolation and Quantitative RT-PCR

Sheep COCs maturated in vitro for 8 h, and the cumulus cells and oocytes of a melatonin group and a control group were then collected, respectively. The cumulus cells and oocytes were washed thrice with DPBS containing 0.1% PVA solution and stored at −80 °C until the RNA was extracted. The total RNA was extracted using a TRizol reagent, quantified by measuring the absorbance at 260 nm and stored at −80 °C until use. The levels of relevant mRNAs, including the oocyte related genes (*GDF9*, *BMP15*, and *DMNT1*), cumulus cells related genes (*PTX3*, *FSHR*, *LHR*, *EGFR*, and *HAS2*) were determined by quantitative RT-PCR using a One Step SYBR PrimeScript RT-PCR Kit (TaKaRa Bio., Inc., Tokyo, Japan) in a Light Cycler instrument (Roche Applied Science, Mannheim, Germany). The levels of accumulated fluorescence were analyzed using the second-derivative method after the melting-curve analysis was completed, and the expression levels of the target genes were then normalized to the expression level of β-actin in each sample. The primer pairs for the analyzed mRNAs are listed in Table 1.

### 4.11. Determination of Intracellular Concentrations of cAMP and cGMP

Intracellular cAMP and cGMP content in oocytes and cumulus cells were determined by a competitive enzyme immunoassay kit (Sigma Chemical Co., St Louis, MO, USA) with an acetylation protocol for the highest test sensitivity according to the manufacturer’s instructions. Briefly, COCs were removed from the culture, denuded, and washed thrice in H-M199 containing 0.2 mM isobutylmethylxanthine (a nonspecific phosphodiesterase inhibitor). The cumulus cells (200 μL 0.1% HCl) and oocytes (1 μL 0.1% HCl/oocyte) were finally collected using 2 mL freezing tubes, snap-frozen in liquid nitrogen for a minimum of 10 min, and stored at −80 °C until assayed. Lysed oocytes and the peroxidase-labeled cAMP or cGMP standards were acetylated using a mixture of triethylamine and acetic anhydride (2:1 *v*/*v*) for 5 min before initiation of the competitive reaction against the anti-cAMP or cGMP antiserum. At the end of the procedure, concentrations of cAMP or cGMP were calculated by measuring the optical density of samples in a plate reader at 405 nm within 30 min. There were 3 samples in each group at least.

### 4.12. Statistical Analysis

In all of the experiments, the data were from at least 3 independent repeats. The data about cumulus expansion, oocyte nuclear maturation, cleavage, and blastocyst rates were converted to percentages. All data were then analyzed in Statistical Product and Service Solutions (SPSS) (version 20.0, SPSS, Inc., Chicago, IL, USA) using a one-way ANOVA with randomized block analysis (linear mixed model) followed by a Tukey test to determine the differences between the treatments. Differences of *p* < 0.05 were considered significant.

## 5. Conclusions

In conclusion, melatonin at the concentration of 10^−7^ M promoted sheep COCs cumulus cells expanding, and increased the cleavage and blastocyst rates of parthenogenetic embryos. MT1 activation in oocytes results in decreased level of cAMP, which inhibits oocyte meiotic mitosis and therefore increases the time of oocyte-cumulus cell gap–junctional communication. The results identified a potentially important role of melatonin in the regulation of sheep oocyte maturation may be primarily mediated by the MT1 receptor.

## Figures and Tables

**Table 1:** Primers used in this study.

Gene Name	Primer Sequence (5′–3′)	Fragment Size (bp)	Reference Sequence Accession Number
*LHR*	TCTGCTCACCCAAGACACTCC	247	XM_005686598.1
	GAGGCAATGAGTAGCAGGTAGAG		
*FSHR*	CTTCCAGAACCTTCCCAACC	201	NM_001285636.1
	TCCCATTCTTACTCAGCCATAC		
*PTX3*	TCTGCGATGGTGTTCTCAGCA	206	XM_005675400.1
	CTCTCTCCTTCAACTGGCGTATG		
*EGFR*	CACTCATGCTCTATGACCCTACCAC	176	XM_005695500.1
	GTGGACACCATCTTCCTCTACCTC		
*GDF9*	AAGGTTCTGTATGATGGGCACG	149	NM_001285708.1
	AGCCGAACAGTGTTGTAGAGGTG		
*BMP15*	CTTCACCTAACTCATTCCCACCTC	248	JQ350891.1
	TGCCACCAGAACTCAAGAACCT		
*DMNT1*	GGACATAATCGGAGATGCTTTGA	206	XM_005688873.1
	AACAGGCTTTGGATGATGAGGT		
*HAS2*	CTTCTCCTGATTCTACGCTTCCT	223	XM_005688874.1
	AACAGGCTTTGGATGATGAGGT		
*GAPDH*	GTGTCTGTTGTGGATCTGACCTG	162	NM_001190390.1
